# How detection ranges and usage stops impact digital contact tracing effectiveness for COVID-19

**DOI:** 10.1038/s41598-021-88768-6

**Published:** 2021-05-03

**Authors:** Konstantin D. Pandl, Scott Thiebes, Manuel Schmidt-Kraepelin, Ali Sunyaev

**Affiliations:** Institute of Applied Informatics and Formal Description Methods, Karlsruhe Institute of Technology, Karlsruhe, Germany

**Keywords:** Biomedical engineering, Electrical and electronic engineering, Computer science, Health policy, Information technology

## Abstract

To combat the COVID-19 pandemic, many countries around the globe have adopted digital contact tracing apps. Various technologies exist to trace contacts that are potentially prone to different types of tracing errors. Here, we study the impact of different proximity detection ranges on the effectiveness and efficiency of digital contact tracing apps. Furthermore, we study a usage stop effect induced by a false positive quarantine. Our results reveal that policy makers should adjust digital contact tracing apps to the behavioral characteristics of a society. Based on this, the proximity detection range should at least cover the range of a disease spread, and be much wider in certain cases. The widely used Bluetooth Low Energy protocol may not necessarily be the most effective technology for contact tracing.

## Introduction

Caused by the SARS-CoV-2 virus, the Coronavirus disease 2019 (COVID-19) has rapidly spread to a global pandemic with more than 136 Million infections and 2.9 Million deaths worldwide, as of April 2021. Only few countries have managed to minimize the number of infections, and many countries struggle to control the epidemic, some facing a second or third, even more severe wave of infections^1^. A widely accepted means to better control the epidemic is the isolation of infectious individuals. To do this effectively, researchers and governments around the globe have discussed and subsequently introduced digital contact tracing (CT) apps. Once such a CT app is installed on someone’s personal device, it automatically tracks and records information about the individual’s proximity to others that have the same CT app installed on their devices. If the individual is diagnosed with COVID-19, this information can be entered into the CT app, which then automatically notifies others that were in physical proximity to the infected person. The notified others thus are informed that they have been exposed to the risk of possible infection and may self-quarantine, test, or take other preventive measures to limit the spread of the disease^2, 3^.

Many countries have developed and implemented their own CT solutions^4^. An important aspect of any CT app implementation is the technique to detect others in proximity. Bluetooth Low Energy, for example, is used in many countries^4^. However, extant research has opposing views about its suitability for CT. On the one hand, it is among the most accurate technologies for CT^5^. On the other hand, lab experiments suggest that a wider distance between smartphones does not necessarily decrease the received signal strength, and Bluetooth Low Energy may register contacts within a relatively wide distance that exceeds the distance in which infections actually happen^6, 7^. Beyond Bluetooth Low Energy, extant research on CT has discussed many other technologies to detect the proximity between individuals such as GPS, QR codes^4^, or RFID^8, 9^ (see Table 1). These technologies vary in several characteristics, like their energy consumption, the time interval of physical distance measurement, or the amount of required user action. With regard to epidemic control, the most important differentiating characteristic of these technologies is the proximity detection range (PDR). While some technologies like RFID detect physical contacts only for small distances, the PDR of Bluetooth Low Energy is up to 10 meters^6^, and sites-wide QR codes provide snapshots of which persons were at a certain place at a certain time. Given these different characteristics, research has recently called to assess and optimize the suitability of proximity detection technologies for CT apps^10^.

**Table 1:** Simulated proximity detection methods and their possible realization with widespread sensors for personal devices.

Proximity detection range	Possible technological realization with personal devices	Exemplary studies or initiatives
0.2 m	Near field communication protocol^11^	Prior literature^12^, no country deployed as of today
1 m	Radio-frequency identification techniques^13^	Prior literature^8^, hospital study in Singapore^14^
	Bluetooth class 3^15^	-
2 m	Bluetooth Low Energy (varying statements in literature, around 2 meters^16, 17^ to up to 10 meters^6^)	CT solutions based on the interface provided by Apple and Google^18^, for example, the Corona-Warn-App from Germany
10 m	Bluetooth class 2^15^	Prior literature^19^
	GPS^20^	HaMagen (Israel), Private Kit (USA)^4^
Sites-wide	QR Codes^21^	Prior literature^21^, ZeroBase (USA)^4^, Luca (Germany), CT solutions in China, England and Wales^22^

In an ideal scenario, CT apps help to detect a contact if an infection event occurred, and do not detect a contact if no infection event occurred. However, in reality no CT technology can achieve this ideal scenario. Instead, the different PDRs of different technologies yield their very own benefits and disadvantages. A PDR wider than a reasonable distance of infection risk may ensure a high recall (i.e., a large share of infectious contacts is registered). However, it may also lead to a low precision (i.e., many of the registered contacts were not actually infected). Such a registration of many contacts may cause large lockdowns. While large lockdowns may be effective to control epidemics, they also yield substantial negative effects such as high economic costs^2, 23^, constraints on personal freedom, and harming the populations’ mental health and well-being^24^. Furthermore, high shares of false positive contacts that unnecessarily (self-)quarantine may lead to low initial adoption rates of CT apps, and even usage stops, thereby potentially decreasing the overall effectiveness of CT. In contrast, a small PDR may lead to the registration of contacts at risk with a high precision, but it may also miss some relevant contacts, and therefore be less effective in controlling the epidemic. Furthermore, a small PDR enables the localization of individuals more accurately and, thus, potentially opens the door to deducing highly precise movement profiles that impede individuals’ privacy. In some regions, reflections on privacy play a key role in the adoption of CT apps. For example, Norway, has stopped its CT app due to public privacy concerns^25^. Given these different considerations, it is not a trivial task for decision makers to decide on the most suitable PDR for CT apps in order to fight the COVID-19 epidemic in their country. Here, we report on a spatial simulation with the aim to analyze how different PDRs implemented in CT apps might influence the course of the COVID-19 epidemic. We thereby also take into account how a usage stop induced by false positive quarantine affects the effectiveness of CT apps for different PDRs.

In the spatial simulation, individuals move in a two-dimensional space and can thereby spread an infectious disease. The parameters of the simulated society are set to closely match the German society, and the infectiousness of COVID-19. Germany is an interesting model country for our simulations for several reasons. First, Germany has taken serious measures on non-mandatory CT, by releasing an app relatively early, with large public investments into the CT app (up to EUR 69 Million until the end of 2021^26^). Second, privacy discussions in general and around CT in particular are very prevalent^27^. Third, Germany has a severe COVID-19 epidemic, in international comparison^1^. Fourth, while Germany was – along with other important measures (e.g., obligations to wear a mask at certain places) – able to drastically reduce the number of COVID-19 cases during the summer of 2020, it is currently confronted with increasing case numbers (commonly referred to as not just the ‘second, but third wave of infections’) and faces the challenge of how existing measures must be adapted in order to fight the epidemic more effectively. Thereby, the effectiveness of the German CT app has recently been questioned in the public discourse, due to its number of downloads of ca. 20.3 Million (as of October 2020)^28^, resulting in a relatively low estimated adoption rate of ca. 24.5%. Furthermore, new alternative CT apps are emerging in local parts of Germany, for example, the Luca app which relies on QR code-based contact tracing at restaurants, bars, or event locations.

Our work contributes to the scientific knowledge base on COVID-19 and the fight against global pandemics via digital technologies in three ways. First, we evaluate the effectiveness of different PDRs for CT in being able to help bringing the pandemic under control under different initial adoption levels. Second, we study the effect of a usage stop, which may occur once individuals stop using CT due to a false positive quarantine. Third, we demonstrate that spatial simulations can provide valuable insights when studying the effectiveness of CT for combating epidemics.

## Results

### Effectiveness of contact tracing

**Figure 1:**
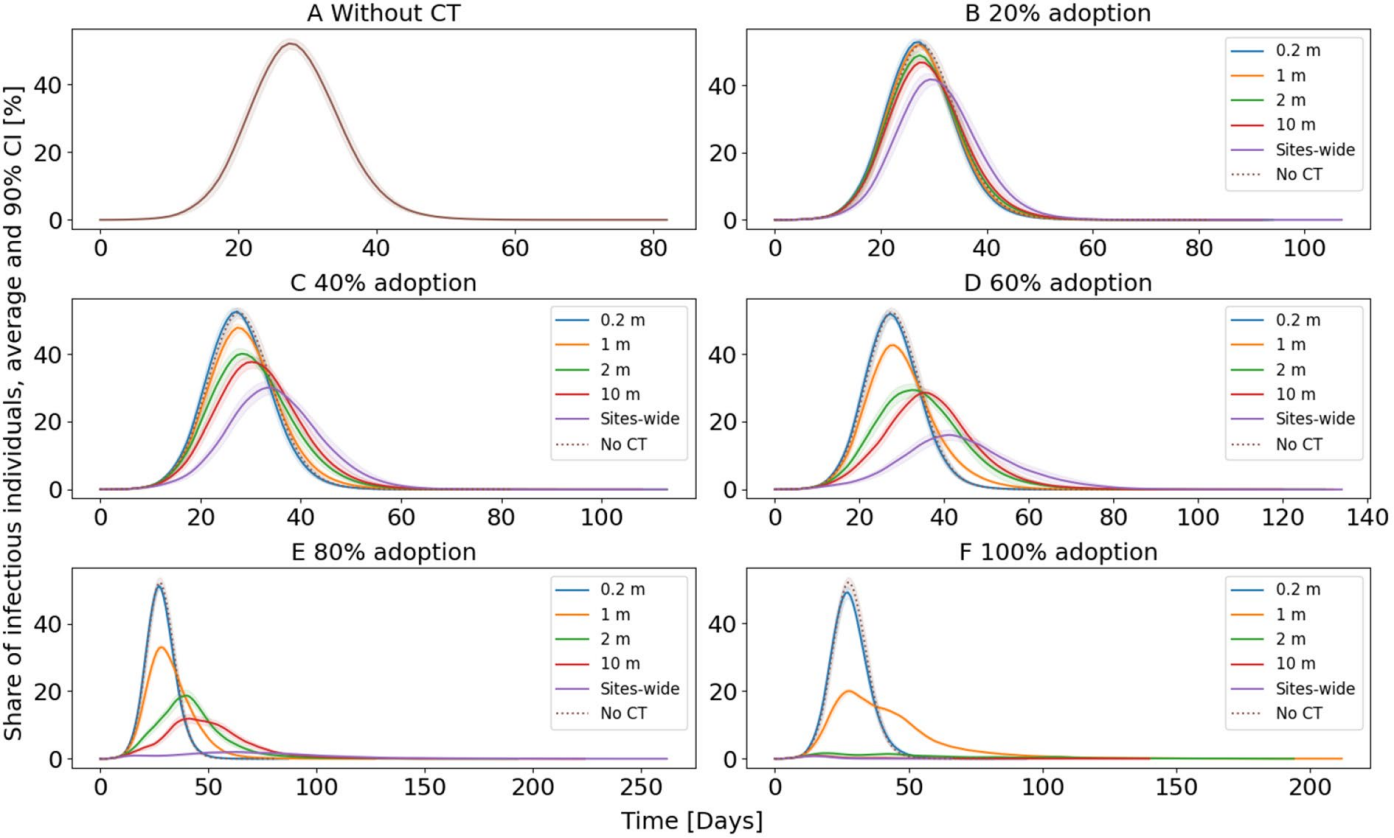
Average share of infectious individuals. For each curve, the mean from 30 simulations, and the 90% confidence intervals (displayed as colored shades behind each curve) are plotted. The characteristics of the individual curves depend highly on the PDR and the adoption rate.

A major goal of controlling the COVID-19 epidemic is to keep the maximum share of infectious individuals low, commonly referred to as ‘flattening the curve’. To analyze how different PDRs of different CT apps might influence the course of the COVID-19 epidemic, we simulated several CT solutions for each adoption level. The course of the average share of infectious individuals over time is illustrated in Fig. 1. In this first simulation, we did not account for usage stops of individuals due to false positive quarantine.

The course of the simulated COVID-19 epidemic without any CT is illustrated in Fig. 1A. The average maximum share of infectious individuals within the 30 simulations is 55.80%. With any CT solution adopted, this maximum decreases. In general, larger PDRs lead to a lower maximum share of infectious individuals than smaller PDRs. The reason for this is that large PDRs are more sensitive and cause more susceptible and infectious individuals to go into quarantine. The susceptible individuals then cannot get infected for the time of the quarantine, whereas the infectious individuals are prevented from infecting others (except for household members). Furthermore, the initial adoption rates of CT apps have a substantial impact on the course of the COVID-19 epidemic. Thereby, higher initial adoption rates lead to lower average maximum shares. For example, for CT based on a 10 m PDR, the average maximum is 50.20% at a 20% adoption rate (see Fig. 1B) and 1.06% at a 100% adoption rate (see Fig. 1F). The lowest maximum average of infectious individuals was 0.79% for the sites-wide PDR with a 100% adoption rate.

**Figure 2:**
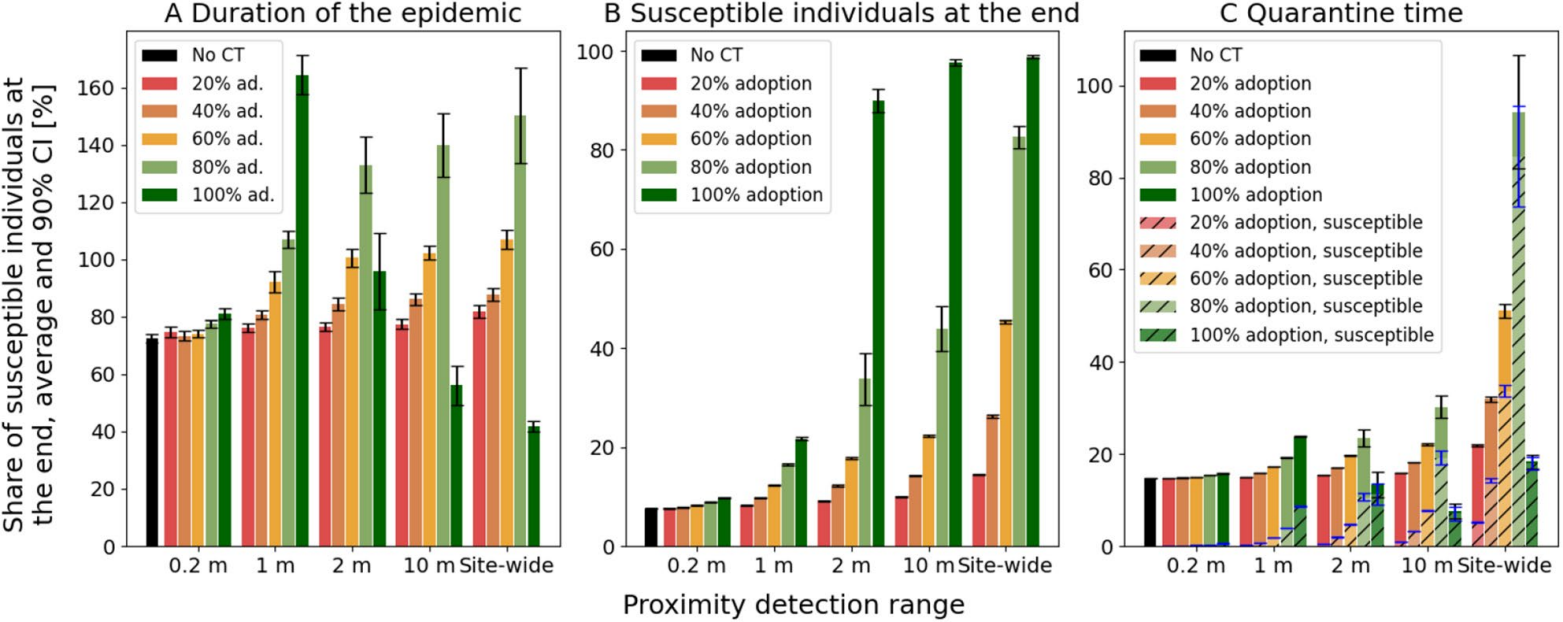
Quarantine time and efficiency measures. Each subfigure illustrates the mean values and 90% confidence intervals based on the used PDRs for CT and the adoption of CT averaged over 30 simulations. **A** shows the duration of the epidemic. For this, Fig. 1 is not representative, as the curves are averaged and, therefore, take the value of the simulation with the longest duration. **B** shows the share of susceptible individuals at the end of the epidemic, **C** shows the average quarantine time per individual, and the average time individuals spend this quarantine time in a susceptible disease state.

Another metric of interest is the duration of the epidemic, which is illustrated in Fig. 2A. Without CT, the average duration was 72.50 days. By flattening the curve, the adoption of CT apps generally led to a prolongation of the epidemic, with a higher adoption rate leading to an increase in the duration. The highest average duration was 164.47 days for a 1 m PDR at a 100% adoption rate. For CT based on PDRs of 2 m or larger, however, higher adoption did not necessarily lead to a prolonged epidemic. CT based on a sites-wide PDR, for example, had the shortest epidemic duration with an average of 41.73 days, followed by CT based on a 10 m PDR with an average of 56.20 days. Both values are shorter than the average duration of the epidemic without CT. The 2 m PDR CT solution adopted at 100% comes with an average epidemic duration of 95.77 days, which is more than the epidemic without CT, but less than the 2 m PDR adopted at 80% (132.97 days).

We further analyzed the share of susceptible individuals at the end of the epidemic, illustrated in Fig. 2B. A high share is preferable, since individuals that never get infected with the disease do not suffer from potential long-term consequences of an infection and will not succumb to the disease. Without any CT, almost all of the individuals in our simulation get infected over time, with the average share of susceptible individuals at the end of the simulation being 7.51%. Conversely, the higher the adoption rate of CT and the larger the PDR for CT, the higher the share of susceptible individuals at the end of the epidemic get. However, the effect of CT with small PDRs (i.e., 0.2 m, 1 m), is limited: With a 1 m PDR CT adopted at 100%, an average of 21.73% of the simulated population is susceptible at the end of the epidemic, whereas a rest (78.27%) has been infected over time. Contrary, a PDR of 2 m at a 100% adoption rate already reaches an average of 89.89% individuals susceptible at the end of the epidemic, and CT with the 10 m PDR reaches even an average of 97.68% susceptible individuals at the end of the epidemic. However, the value of the 10 m PDR CT is only 43.88% when adopted at 80%. With CT based on a sites-wide PDR, the value at 80% adoption rate is 82.57% and 98.82% at 100% adoption rate.

A major cost of CT is quarantine time, which can have significant negative effects, such as economic losses^2, 23^, strains on mental health^24, 29^, untreated medical conditions^30^, or increases in domestic violence^31^. Therefore, we analyzed the average quarantine time per person, which is illustrated in Fig. 2C. Without CT, the average quarantine time per person was 14.69 days, due to quarantined infectious individuals, and their household members. In general, the average quarantine time per person increased with a higher CT adoption rate and larger PDRs. It reaches its maximum at 94.28 days for the sites-wide PDR with a 80% adoption rate. However, with adoption rates above 80%, CT based on a 2 m PDR, a 10 m PDR, or sites-wide PDR showed less quarantine time. At an adoption rate of 100%, the average quarantine time thereby is the lowest for CT based on a 10 m PDR with 7.55 days. CT based on a 2 m PDR resulted in 13.40 days, and CT based on sites-wide PDR resulted in 18.32 days of average quarantine time, which is slightly higher than the epidemic without any CT. Based on the average quarantine duration per person, we also analyzed what share of this time was spent in a susceptible state. In general, quarantine measures aim to target infectious individuals. It also seems reasonable to quarantine exposed individuals, as they may become infectious any time, and in practice it may be challenging to diagnose whether an individual is exposed but not infectious. However, one could consider a quarantine of susceptible individuals as false positive quarantine. The results of our analysis are illustrated in Fig. 2C as hatched bars. Without CT, there are no susceptible individuals in quarantine, since only individuals with symptoms, which are also infectious, and their exposed household members go into quarantine. With CT, also susceptible individuals go into quarantine. For all PDRs, a higher adoption rate of CT leads to a higher share of susceptible individuals in quarantine. Furthermore, CT based on PDRs with a wider detection radius generally lead to a higher share of quarantine time spent in a susceptible state, as they register contacts at a relatively far away distance, where an actual infection event is unlikely to occur. The highest share of time spent in quarantine by susceptible individuals was 98.49% with CT based on the sites-wide PDR adopted at 100%. With a 2 m PDR based CT adopted at 100%, the share was still 85.15%.

### Contact tracing with a usage stop

After being in a quarantine and not feeling symptoms of sickness, an individual may perceive their quarantine as unnecessary. Eventually, quarantines perceived as unnecessary may, among other reasons (e.g., privacy concerns, general mistrust in a CT app or the government), lead to individuals stopping to use the CT app and a lower overall adoption rate of CT apps, thus impeding the effectiveness of CT. To account for this effect, we simulated usage stops after a false positive quarantine with different probabilities (25%, 50%, 75%, or 100%). In these simulations, we assumed an initial CT adoption rate of 60%. This is the recommended minimum adoption rate in other extant research^2^. At the same time, it appears as realistic in western societies, as a 2015 US-based survey revealed that 58.23% of mobile phone users had downloaded a health-related mobile app^32^ and it is likely to assume that the smartphone adoption and health app usage has increased since then.

**Figure 3:**
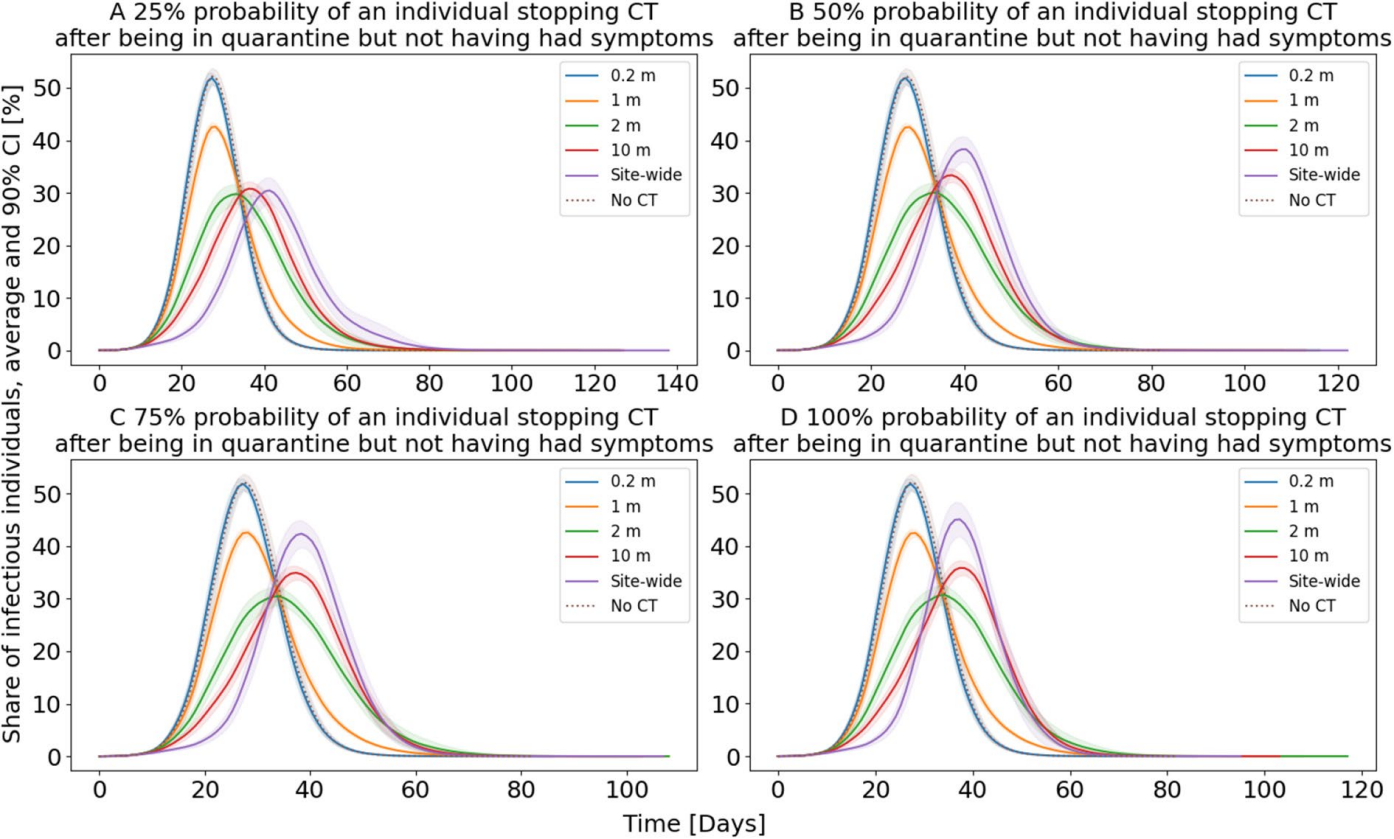
Usage stop of CT after being in a false positive quarantine. For each curve, the mean from 30 simulations, and the 90% confidence intervals are plotted. Thereby, the initial adoption rate of CT is 60%. The probability of stopping CT after a false positive quarantine varies in the subfigures.

The results of these simulations are illustrated in Fig. 3. Similar to the analysis conducted for Fig. 1, we focus on the average share of infectious individuals over time. For CT with small PDRs (0.2 m, 1 m), the effect of this usage stop is almost non-existent. Without any usage stop, the average maximum was 54.71%, respectively 44.28%. With a 100% probability of a usage stop, these maxima are 54.72% and 44.26%. The small increase for the 0.2 m PDR is likely due to a statistical variation and well within the 90% confidence interval. For CT based on a 2 m PDR with no usage stop, the average maximum is 32.49%. This value only slightly increases to up to 33.54% with a 100% probability usage stop. One reason for this observation may be that CT based on short PDR produces only very little false positives, and, thus, only very few individuals stopped using the CT app in our simulation. For CT based on larger PDRs, however, the usage stop has a severe effect on the average maximum share of infectious individuals. Without a usage stop, the values are 30.68% for a 10 m PDR and 19.48% for sites-wide PDR at 60% initial adoption. When studying a 25% probability of a usage stop, the 10 m PDR and the sites-wide PDR come with a 33.50% respectively 35.85% average maximum, thus being similarly effective than the 2 m PDR with 32.54%. This trend intensifies with an increasing probability of the usage stop, where especially the 10 m PDR and the sites-wide PDR become less effective. At a 100% probability, the CT based on sites-wide PDR comes with an average maximum share of 52.27% and is, thus, higher than CT based on a 2 m PDR with 33.54% and even higher than CT based on a 1 m PDR at 44.26%. CT based on a 10 m PDR lies in the middle of the latter two with 38.55%.

## Discussion

With the simulation, we studied how various CT solutions based on different PDRs affect the course of an epidemic. Generally, the adoption of CT helped in all simulations to control the epidemic in the sense of decreasing the maximum share of infectious individuals, and increasing the share of susceptible individuals at the end of the epidemic. In doing so, introducing a CT app also helped to decrease the R_0_ value of the infectious disease within our simulation (see Table S2 in the supplementary information for detailed information on R_0_ values). Our results are in line with previous research suggesting that high adoption rates of CT are beneficial^21^. While our results confirm prior results that a certain adoption rate is necessary to effectively stop an epidemic right from the beginning^2^, our research also suggests that there is no minimum threshold of CT to be effective, but that instead every participation can help to better control the epidemic and increase the effectiveness of CT itself.

The results of our spatial simulations show that each of the different PDRs yields its own advantages and disadvantages when it comes to fighting the COVID-19 epidemic. We summarize these advantages and disadvantages in Table 2. In general, a 0.2 m or 1 m detection range appear to only have limited effectiveness, illustrated, for example, by the share of susceptible individuals at the end of the pandemic, which is only 36.60% on average for the 1 m PDR even at 100% adoption rate. The 2 m, 10 m, and sites-wide PDR are all very effective in controlling the epidemic at 100% adoption rate. The sites-wide PDR is even highly effective at only 80% adoption rate. However, this effectiveness comes with several disadvantages such as very long quarantine duration, and high shares of susceptible individuals in quarantine (false positives). For 100% adoption rate of CT with a 10 m or sites-wide PDR, the average R_0_ value was actually below 1, meaning an infectious individual on average infects less than one other individual. Thus, both CT solutions enabled a declining epidemic right from the beginning. In all other simulation settings, the average R_0_ value always was above the critical threshold of 1, meaning not only CT, but also a growing herd immunity enabled an eventual decline of the epidemic. When simulating a usage stop with an initial adoption of 60%, the advantages and disadvantages of different PDRs for CT substantially changed. PDRs with a short detection range (0.2 m, 1 m) were unaffected, however, PDRs with a wide detection range (10 m, sites-wide) performed much worse. Under this scenario, our simulations demonstrated that the most effective CT was based on a 2 m PDR since the 10 m and the sites-wide PDR showed higher average maximum shares of infectious individuals and, thus, were less effective in ‘flattening the curve’.

**Table 2:** Simulated PDRs and their advantages and disadvantages for CT.

PDR	Advantages	Disadvantages
0.2 m	• Lowest risk of detecting false positives	• Almost no positive impact on maximum share of infectious individuals, or share of susceptible individuals at the epidemic end
1 m	• Noticeable decrease of the maximum share of infectious individuals at high adoption rates • Relatively little quarantine time required • Little share of susceptible individuals in quarantine	• Relatively low share of susceptible individuals at the end of the epidemic, even at 100% adoption rate • Long epidemic duration at 100% adoption rate
2 m	• Highly effective for epidemic control at 100% adoption rate • Relatively little quarantine time required • The most effective PDR for a 60% adoption rate with a usage stop	• Relatively high share of susceptible individuals in quarantine at 100% adoption • Much less effective at lower adoption rates compared to sites-wide CT
10 m	• Highly effective for epidemic control at 100% adoption rate • The least amount of quarantine time required of all PDRs at 100% adoption rate	• Relatively high share of susceptible individuals in quarantine at an 80% adoption rate or higher • Loses effectiveness under a scenario of a usage stop because of false positive quarantines, even at just 25% probability
Sites-wide	• Highly effective for epidemic control, when considering the maximum share of infectious individuals and the share of susceptible individuals at the end, even at 80% adoption rate • Out of all PDRs, the lowest epidemic duration at 100% adoption rate	• Highest share of quarantine time by susceptible individuals of more than 50% at only 60% adoption rate or higher • Relatively long quarantine times at an adoption rate of 60% or 80% • Strongly loses its effectiveness at 60% initial adoption and a usage stop effect

Our results imply that there is no silver bullet CT technology for fighting the SARS-CoV-2 epidemic. Instead, choosing the right CT technology always means finding a compromise between various factors such as effectiveness in controlling the epidemic, false positive quarantine, share of susceptible people, and privacy concerns. In this regard, our work can help decision makers to assess the advantages and disadvantages of the different technologies and, thus, make more informed decisions. Different countries with different cultures have shown widely varying acceptance rates of COVID-19 measures in general (e.g., lockdown, mask requirements), and varying adoption rates of CT apps in particular. Factors influencing the adoption rates may include privacy concerns, the perception of the CT app’s effectiveness, and the perceived quarantine time required to comply with CT apps. Furthermore, some CT technologies may be easy to enforce (e.g., scanning a QR code before entering places of everyday life), which could increase the adoption rate and effectiveness of a CT app, compared to other CT technologies. In some countries, CT based on wide PDRs (i.e., 10 m, sites-wide) may have higher adoption rates than CT based on narrower PDRs due to their higher effectiveness, high acceptance of false positive quarantine, or their absence of causing highly precise movement profiles. Bluetooth Class 2, GPS, wider versions of Bluetooth Low Energy, or sites-wide QR codes may be appropriate technologies for CT apps in such countries (see Table 1). In other countries, however, there may be a high chance of a usage stop after a false positive quarantine. In such cases, our results support the adoption of PDRs that have at least a range of a typical disease spread, but not much more. Bluetooth Low Energy may be an appropriate technology for CT apps in such countries. The results of our simulations also show that technologies with very short PDRs such as Near-Field Communication are not suitable for CT in the case of the COVID-19 epidemic since they resulted in almost no positive impact on the maximum share of infectious individuals, or share of susceptible individuals at the end. To stimulate the public debate on CT for COVID-19 and for potential future pandemics, and to ease drawing conclusions from this article, we summarize the key findings and implications for the public and policy makers in Table 3.

**Table 3:** Key findings and implications for the public and policy makers of our article.

Key findings	Implications for the public and policy makers
The adoption of CT—even if low—always helps to dampen the spread of the epidemic, and a higher adoption rate of CT is more effective thereby	Provide a CT solution in case of an epidemic
	Aim for a high adoption rate of the CT solution by informing the public about the necessity of a high adoption rate
Very short PDRs are not suitable for CT in the case of the COVID-19 epidemic	Use proximity detection techniques with a range of at least the distance of disease spread, depending on the epidemic. For COVID-19, technologies such as Bluetooth Low Energy, GPS, or QR-codes are suitable
Usage stops can severely impact the effectiveness of CT in the long run, especially for wide PDRs	Clarify the probability of false positive contacts, and the likelihood of usage stops following it
	If the potential for usage stops after false-positive quarantines is generally high in society (e.g., larger than 25%), consider using a combination of proximity detection techniques (i.e., wide range and medium range techniques) adapted to the epidemic situation and society to keep the number of false-positive quarantines and following usage stops low
	If the potential for usage stops after false-positive quarantines is generally low in society, consider using a wide range proximity detection technique to detect as many infectious contacts as possible and thereby minimize the number of unquarantined infectious individuals
The ideal approach for CT depends on many different factors. The example of the case of Germany shows that immature CT solutions may be rejected by the society, and salvaging public’s interest is challenging	Though the selection of the CT mechanism has to pass off quickly, different approaches need to be thoughtfully considered and the most promising should be chosen based on multiple factors (e.g., transmissibility of the infectious disease, mobility of individuals, closeness of contacts, acceptable duration of quarantine, acceptance of false positive contacts)
	Factors influencing the effectiveness of CT highly depend on cultural and societal aspects found in individual regions. Simulation methods can help to design and evaluate fitting CT mechanisms quickly and at the beginnings of an epidemic

With our work, we follow calls for research on the technical design of CT apps^10^ and open multiple opportunities for future research. However, our work is limited by some factors as the real world is always more complex than a simulation. For example, our simulation especially accounted for droplet infection, where CT can be highly effective. Meanwhile research suggest that aerosol transmission or fomite transmission are another plausible mode of SARS-CoV-2 transmission^33^. As these modes of transmission do not directly depend on the physical proximity between individuals, CT may likely be less effective for epidemic control in such scenarios. Future research should aim to incorporate these factors into more complex simulation models. Furthermore, contact patterns are more complex in the real world than in our simulations. Beyond schools, workplaces, and supermarkets, there are many more locations where infections can likely occur, for example, at restaurants, bars, other shops, or public transport. The spatial architecture and movement patterns of such other places are different to the ones in our simulation. For example, people come into closer contact with each other when many individuals enter or leave public transport, and restaurants and bars have waiters who come into contact with many spatially distributed individuals. While the general principle of CT still remains effective when considering such a broader variety of locations, the dynamics of the simulated epidemic and the effects of CT adoption on the course of the epidemic may change (e.g., a dense accumulation of individuals when entering or exiting public transport may further spread the infectious disease, but may also lead to more false-positive traced contacts for medium to wide-range PDRs, which itself may lead to a lower long-term adoption rate, and thus, a reinforced epidemic). Additionally, our simulation does not account for a varying adoption rate of CT and varying mobility of individuals depending on the age of the individuals. In practice, older individuals could be less likely to use a mobile device and CT but may also be likely to have a lower mobility, whereas younger individuals could be more likely to adopt CT, and also have a higher mobility. Future research should, therefore, aim to develop more accurate measures for contact patterns and CT usage patterns (e.g., depending on the age) for such spatial simulations. We made the source code of our simulations publicly available for such purposes.

In general, the research on SARS-CoV-2 transmission is still ongoing. For our simulations, we required a probability distribution which tells us the likelihood of an infection, depending on the distance between the infectious and a susceptible individual. Our most likely choice was a half normal distribution which we parametrized such that we obtained an R_0_ value realistic for COVID-19. While we believe our choice is reasonable, future research should aim to further quantify this probability distribution with real-world evidence. However, such viral experiments may be challenging to conduct. Moreover, recent research suggests that already recovered individuals can get infected with SARS-CoV-2 for a second time. Therefore, another interesting extension of our work would be the use of a SEIRS model instead of a SEIR model, where individuals can transition back to a susceptible state from the recovered state. Likewise, our current model does not account for deaths or hospitalizations. While accounting for deaths in an adapted simulation model does not change the course of the epidemic or the effectiveness of CT per se (i.e., dead individuals are less likely to infect other individuals, similar to recovered individuals), it is still a very important metric to observe. Illustrating the potential of CT to prevent deaths through future simulation studies may help to increase CT adoption and ultimately effectiveness. Finally, an interesting field for future research may be the use of different PDRs at different places, for example, depending on the location type or room size. Depending on different transmission dynamics at different places, different PDRs may be optimal at different types of places, and an adaptive PDR selection strategy may provide better overall results.

Extant research has demonstrated that the economic and societal consequences of large lockdowns with many quarantined people are highly severe. In this regard, we demonstrate that choosing the right CT technology with a suitable PDR can play a key role in determining whether large lockdowns become necessary or not. On the one hand, short PDRs are limited in their effectiveness of fighting the epidemic since they potentially miss to track important contacts. On the other hand, wide PDRs should also be selected with care since they lead to high shares of false positive quarantine. Our results suggest, that for many scenarios, the most promising CT solutions are based on a PDR that roughly corresponds to the infection range. However, this finding is dependent on various different factors such as initial adoption rate, and potential usage stops. Consequently, we recommend further exploration of reasons for such usage stops and means to ensure extensive continuous use of CT apps. Within a broader picture, effective CT for COVID-19 demonstrates the potential of digital technologies in tackling future challenges for health care systems around the world.

## Methods

In the following, we provide an overview over our simulation method. For a more detailed description, please see the supplementary information. For the simulations, we followed prior epidemiologic research and evidence where infections are likely to occur^34^, and implemented four types of places in order to obtain a mixture of everyday social situations: households, schools, workplaces, and supermarkets. The parameters of our simulations are set such that the base reproduction number R_0_ of the pandemic, without contact tracing measures, has a median of 2.792, which is close to a median value of 2.79 that extant research has identified for the COVID-19 pandemic^35^.

**Figure 4:**
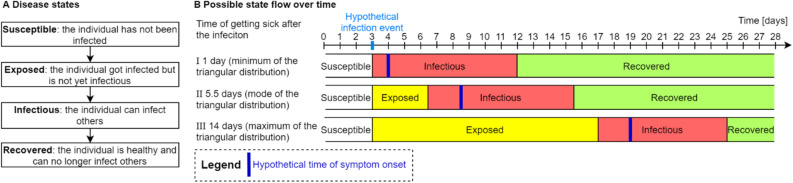
**A** shows the disease states and flow of the SEIR model. **B** shows different cases (minimum, mode, and maximum of the triangular distribution) on a time line.

The epidemic in the simulations builds on the SEIR model, which states that an individual can be in one of four states: susceptible, exposed, infectious, or recovered^36^. Initially, almost all individuals are in a susceptible state, and few are exposed to the infectious disease. Exposed individuals then transition into the infectious state after a certain time, in which they can infect other susceptible individuals. After some time, an infectious individual recovers and stays immune to the infectious disease. This flow of states is also illustrated in Fig. 4. Prior research showed that the SEIR model is well applicable for COVID-19^37^. At the start of a simulation, ten individuals are exposed, whereas the rest of the population is susceptible. An exposed individual then develops symptoms after the incubation period, which we modeled through a triangular distribution with a minimum of 1 day, a mode of 5.5 days, and a maximum of 14 days, representing a realistic value for COVID-19^38^. However, individuals already transmit COVID-19 around two days before they develop symptoms^39^. Accordingly, individuals in the exposed state transition into the infectious state two days before they develop symptoms in our model. Once individuals develop symptoms, we presume that they and their household members go into quarantine for 14 days, the household structure thereby is similar to the German household structure^40^ (see supplementary information for detailed information on the household structure). Since the COVID-19 infectiousness typically declines within seven days after symptom onset^39^, individuals remain in the infectious state for nine days, before transitioning into the recovered state. Infectious individuals can infect susceptible individuals within a 2-m distance. The likelihood of an infection event depends on the distance between an infectious and a susceptible individual and follows a half normal distribution (i.e., the smaller the distance, the more likely an infection event). Within households, we presume that an infectious family member always infects other members, as individuals within a household typically are within less than 2-m distance for an extended period of time.

**Figure 5:**
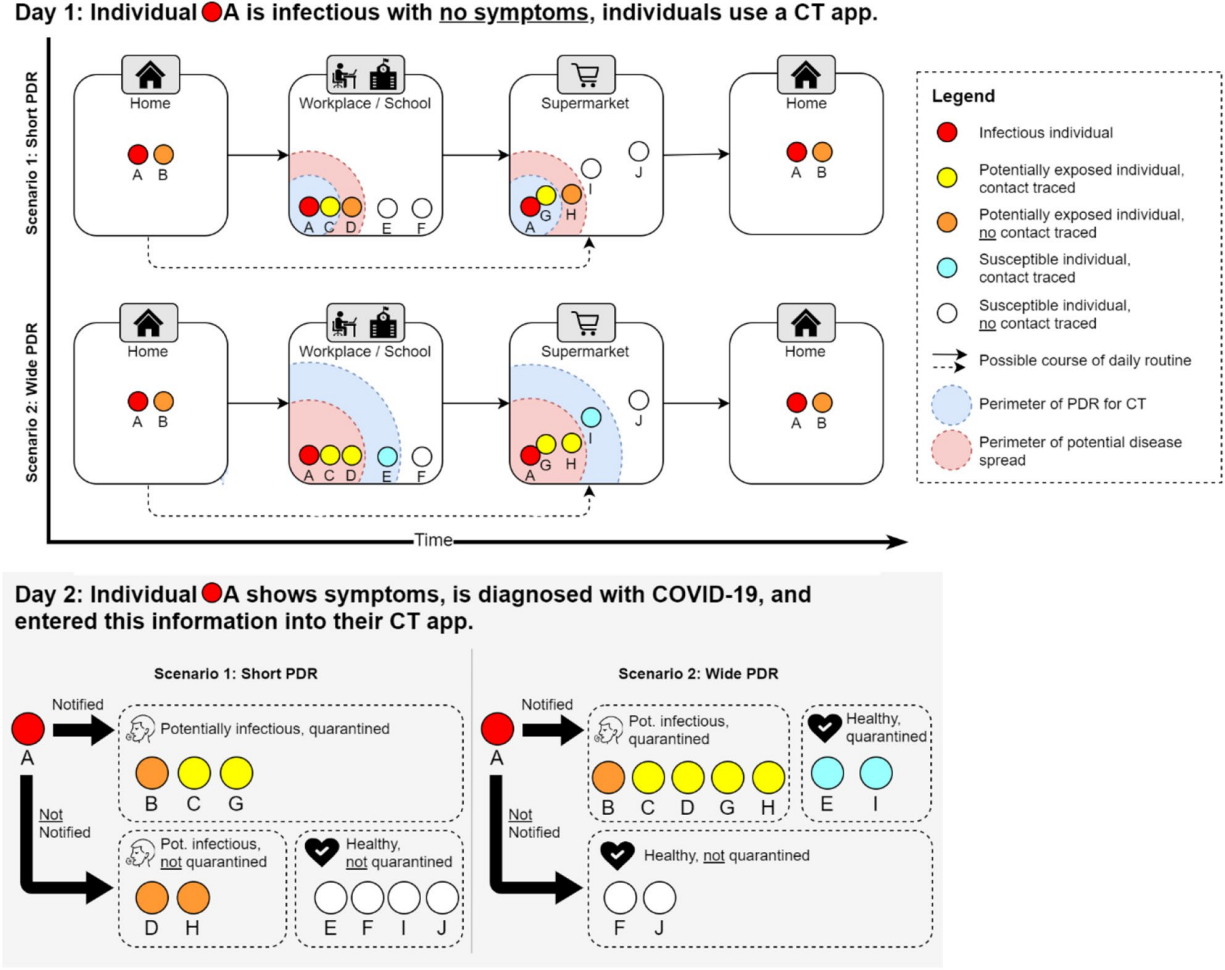
Illustration of the simulation of CT apps with a small PDR, and a wide PDR. On day one, individual A is infectious but does not yet show symptoms. Therefore, individual A follows a daily routine and infects other individuals. Depending on the PDR of the CT apps, different contacts with other individuals are traced. At the end of day one, individual A develops symptoms and, thus, contacts are notified. On day two, individual A and the contacts go into quarantine, while non-contacts follow their daily routine.

We also simulated the epidemic when CT solutions are applied. Overall, we simulated 46 different scenarios. These scenarios differ in the CT adoption level, the PDR for CT, and the probability of individuals stopping to use the CT app after a false positive quarantine. The initial CT adoption levels in our simulation scenarios are 20%, 40%, 60%, 80%, or 100% of the simulated population. Within each of these initial adoption levels, we simulated five different PDRs for CT apps. For four of the CT apps to register a contact, individuals need to be within a certain range for at least 15 s, specifically of 0.2, 1, 2, or 10-m. For the fifth simulated CT app, individuals scan a sites-wide QR code provided at a location (i.e., workplace, school, supermarket) which changes once a day. In practice, each of these PDRs can be realized with sensors built-in in widely available smartphones. Table 1 provides an overview of possible realizations. We did not trace contacts within households, as we presume that household members communicate and quarantine in case another household member shows symptoms. To account for possible usage stops in case of false-positive quarantine, we conducted simulations with individuals stopping CT usage with a probability of either 25%, 50%, 75%, or 100% in case they were falsely quarantined. We simulated each scenario 30 times with different random seeds to obtain averaged results. Each simulation contained a society of 10,000 individuals per scenario and a time step of 100 ms. The code and random seeds we used for our simulation are available on GitHub, thus allowing other researchers to verify and build on our results^41^. Figure 5 exemplifies typical 2-day routines within our spatial simulation when an infection occurs on day one. Two example scenarios (short PDR and wide PDR) are illustrated. In both scenarios, the presence of a CT app potentially leads to wrong conclusions about individuals’ health status. In the short PDR scenario, the individuals D and H are potentially infectious but not tracked by CT (false negative) and in the wide PDR scenario, the individuals E and I are healthy but tracked by CT and subsequently quarantined (false positive).

## Supplementary Information

Supplementary Information

## Data Availability

The source code for the simulation is available on GitHub^41^, allowing researchers to build upon our simulation. Random number generators are seeded, allowing researchers to reproduce our results. For more details on the simulation method, please also see the supplementary information.
