# Supplementary material for: How detection ranges and usage stops impact digital contact tracing effectiveness for COVID-19
Source: Sci Rep. 2021 May 3;11:9414. doi: 10.1038/s41598-021-88768-6 (PMC8093197; doi:10.1038/s41598-021-88768-6)
Supplement: Supplementary file 1 — Supplementary Information [file 41598_2021_88768_MOESM1_ESM.pdf]

Supplementary information

How detection ranges and usage stops impact digital contact tracing effectiveness for COVID-19

Konstantin D. Pandl[1], Scott Thiebes[1], Manuel Schmidt-Kraepelin[1], and Ali Sunyaev[1,*]

[1]Institute of Applied Informatics and Formal Description Methods, Karlsruhe Institute of Technology, Karlsruhe, Germany
[*]sunyaev@kit.edu

Materials and methods

General simulation model

Our simulation model consists of a population of 10,000 individuals living in households. The household size distribution follows the German household size distribution (1) and is illustrated in Table S1. We assume that individuals return to their household every day.

Table S1. Household sizes and their share of the total number of households within the simulation.

Household size [number of individuals]	Share of total number of households [%]
1	42.3
2	33.2
3	11.9
4	9.1
5	3.5

Following prior work on the spread of infectious diseases (2), individuals can meet at four different types of places. In our simulation, these places are workplaces, schools, supermarkets, and households. Similar to the German society, we assumed that 70.1% of the population regularly visit a workplace or school (including universities and kindergartens). This estimated share is based on ca. 44.7 million individuals working (3), ca. 8.3 million pupils in school (4), ca. 2.8 million university students (5), and 2.4 million kindergarteners (6), out of a German population of 83 million individuals. In our simulation, these workplaces or schools have a quadratic shape (as illustrated in Figure S1 B) with a row capacity between 2 and 8, thus, the total capacity of the specific location is between 4 and 64. When visiting a workplace or school, an individual randomly selects one seat, which ensures a mixing of different contacts in our simulation. The distance between each row and column for a workplace or school are randomly sampled between 1.5 and 2-meters. Thereby, individuals can move along aisles in order to go to their seats with a constant speed of 1 $\frac{m}{s}$. Individuals arrive between 8 AM and 8:30 AM and stay there for 8 hours.

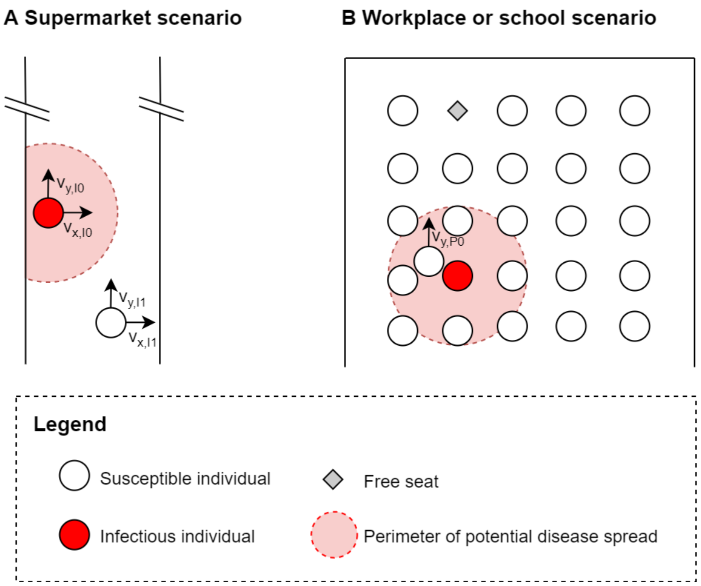

Fig. S1. Illustration of the spatial simulation locations. Besides households, infectious individuals can infect other individuals at supermarkets, workplaces, and schools. This figure shows individuals at these places, some of the shown individuals are moving, illustrated by a speed component in x and y direction. Specifically, A shows a supermarket scenario, where individuals move along an aisle. B shows a workplace or school scenario in quadratic shape, where an individual moves to a randomly assigned seat when arriving, and may get infected while doing so.

If an individual is not at their workplace or school, they can visit a supermarket once a day with a daily probability of 20%. These are open Monday through Saturday from 8 AM till 8 PM. For the 10,000 individuals in the population, there are three supermarkets, resulting in a comparable distribution of supermarkets per capita to Germany (7). A supermarket is simulated as one long aisle, as illustrated in Figure S1 A. Individuals can go in both directions, forward, and backward, which increases the mixture of contacts. To do so, each individual has a constant base speed in y direction, which is determined such that the planned duration of the visit is a random number between 15 and 45 minutes. To allow individuals walking backwards, an individual walks with an acceleration in y direction which takes a sinusoidal form. The period duration is three minutes, the amplitude is determined such that the maximum additional speed gain from this sinusoidal function is the difference to the maximum speed of 1 $\frac{m}{s}$. However, this maximum additional speed gain is divided by a random integer between 1 and 10. As a result, some individuals occasionally walk backward (meaning they walk with a negative speed), whereas others walk almost with a straight speed. The x acceleration is determined randomly, but capped such that the maximum total speed (consisting of a speed in x direction and a speed in y direction) is 1 m/s. Furthermore, the individuals always stay within the physical boundaries.

In general, the next position of a person as a vector is determined by its current speed $\vec{v}$, its acceleration $\vec{a}$, and the simulation time step Δt:

$$\begin{pmatrix} X_{new} \\ Y_{new} \end{pmatrix} = \begin{pmatrix} X_{prior} + v_X * \Delta t + 0.5 \ * \ a_X * \Delta t^2 \\ Y_{prior} + v_Y * \Delta t + 0.5 \ * \ a_Y * \Delta t^2 \end{pmatrix}$$

The speed vector of the person after the next time step is defined as follows:

$$\begin{pmatrix} v_{x,new} \\ v_{y,new} \end{pmatrix} = \begin{pmatrix} v_{x,prior} + a_X * \Delta t \\ v_{y,prior} + a_Y * \Delta t \end{pmatrix}$$

The time step in our simulation Δt is 100 ms, as this allows us to simulate walking speeds in the order of about 1 $\frac{m}{s}$ and measure distances in the order of decimeters at the same time.

Epidemic simulation

Another essential part of our simulation model is the infectious disease. We aim to model this infectious disease realistic to COVID-19 with a SEIR model, where individuals are either in a susceptible, an exposed, an infectious, or a recovered state. This flow of states is also illustrated in Figure 4 of the main article.

At the start of the simulation, ten individuals of the population are exposed and the rest of the population is susceptible. The transition of a susceptible state to an exposed state occurs when an individual gets infected. Afterwards, it takes a certain duration until the individual develops symptoms. In accordance with extant knowledge on COVID-19 (8), we model this duration as a random number sampled from a triangular distribution with a minimum of 1 day, a mode of 5.5 days, and a maximum of 14 days. Similarly, two days before an individual develops symptoms, its disease status transits from the exposed state to the infectious state. However, the duration in the exposed state cannot be negative. The duration in the infectious state is 9 days, effectively meaning 2 days before the symptoms and 7 days after the symptoms are noticed (9). We implemented the spatial simulation for the workplaces, schools, and supermarkets, but did not implement it for the households. Instead, we assume that household members typically have a close contact between each other, therefore, in the simulation model an individual infects other household members once they are infectious. Furthermore, an individual and their household members go into quarantine the day after an individual showed symptoms.

A further important set of parameters determines the probability of an infection event. For such an infection event, an infectious and a susceptible individual need to be within a certain distance and the closer they are, the more likely the infection event occurs. Specifically, an infection event can occur only if the two individuals are in proximity of 2 m or closer. In our simulation, each infectious individual is assigned a fixed random value σ from a triangular distribution with a minimum of 0, a mode of 0.5 m, and a maximum of 1 m. This value accounts for a varying infectiousness between individuals which is observed for COVID-19 (10). The higher it is, the more infectious an individual is, given it is in the infectious state of the disease. Once a susceptible individual is in proximity of an infectious individual, a random number from a half normal distribution is drawn. This half normal distribution has a standard deviation of σ from the infectious individual. An infection event can then occur if the distance between the two individuals is smaller than this drawn random number. This mechanism alone, however, would lead many more infections if the simulation time step Δt is small (it is 100 ms in our simulations). Therefore, another condition has to be met: a random event of 5% probability needs

to be drawn to be true. The first condition ensures that an infection event is more likely, the closer two individuals are. A half normal distribution was chosen instead of a normal distribution because the distance between individuals is always a non-negative value. The second condition is necessary in order to adapt the likelihood of an infection to the simulation time step. Based on 30 simulations without any CT, this setting results in in an average base reproduction number of R_0 = 2.799, a median of R_0 = 2.792, and a 90% confidence interval between 2.787 and 2.811. The median value in our simulation was close to the median value of 2.79 that was identified for the SARS-CoV-2 pandemic by extant research (11).

Measurement of the base reproduction rate

As discussed above, we set a few parameters such that we obtain a reasonable value for the base reproduction number R_0. This affects parameters that define the likelihood of an infection, given an infectious individual is in proximity of a susceptible individual. This section describes how we calculated the base reproduction rate R_0 out of our simulation results.

In a simplified SEIR model (12, 13) with a natural death rate of $\mu = 0$, the base reproduction rate is given by $R_0 = \frac{\beta}{\gamma}$. Thereby, γ is the reciprocal of the infectious period and β is the effective contact rate. The SEIR model is described by a system of differential equations, which themselves describe the change of four quantities. These four quantities are the number of susceptible individuals at a given time $S(t)$, as well as the number of exposed individuals $E(t)$, the number of infectious individuals $I(t)$, and the number of recovered individuals at a given time $R(t)$. The system of differential equations is as follows (N is the size of the population, the average incubation period is a^{-1}):

$$\frac{dS(t)}{dt} = -\beta S(t)\frac{I(t)}{N}$$
$$\frac{dE(t)}{dt} = \beta S(t)\frac{I(t)}{N} - aE(t)$$
$$\frac{dI(t)}{dt} = aE(t) - \gamma I(t)$$
$$\frac{dR(t)}{dt} = \gamma I(t)$$

By dividing $\frac{dR(t)}{dS(t)}$, we obtain $\frac{dR(t)}{dS(t)} = -\frac{\gamma N}{\beta S(t)} = -\frac{N}{S(t)R_0}$. We then integrate from the start of the simulation ($t = 0$) to the end of the simulation ($t = \infty$) and obtain $R(\infty) - R(0) = \left[\frac{-N}{R_0} ln(S(t))\right]_0^\infty = \frac{-N}{R_0} ln(S(\infty)) + \frac{N}{R_0} ln(S(0)) = \frac{N}{R_0}(ln(S(0)) - ln(S(\infty)))$. With $R(\infty) = N - S(\infty)$ and $R(0) = 0$, we obtain $N - S(\infty) = \frac{N}{R_0}(ln(S(0)) - ln(S(\infty)))$, and ultimately, we obtain $R_0 = \frac{N}{N-S(\infty)}(ln(S(0) - ln(S(\infty))$. Thus, we can calculate R_0 by the number of individuals in our simulated population $N = 10{,}000$, the number of initially susceptible individuals $S(0) = 9{,}990$, and the number of susceptible individuals at the end of the simulation $S(\infty)$.

Simulation process

We simulated on a high-performance computing cluster with nodes consisting of an Intel Xeon Gold 6230 CPU at 2.1 GHz frequency. In total, we simulated 46 different types of scenarios (composed of different CT adoption rates, different PDRs, and varying probabilities of a usage stop) Each scenario type was simulated 30 times (with different random seeds), in order to obtain smoothed means and confidence intervals. Each simulation ran on one CPU core, therefore, we computed with 1,380 CPU cores in total. Depending on the simulation parameters and random influences, the simulation time varied, most of the simulations finished within 1 and 2 days, the longest one took ca. 7.8 days. In order to decrease the required simulation time, one could increase the simulation time step Δt (e.g., to 1 s instead of 100 ms), and one could reduce the size of population. However, this can lead to less accurate results, and a higher variance; this is the reason we set the simulation time step to 100 ms. The source code to simulate and visualize the results is available at (14).

Simulation results

Table S2 shows the main results of our study with the mean value and 90 % confidence intervals based on 30 simulations. Most of these results are also visualized in Figure 2, 3, and 4 within the main article.

Table S2. Results of the different simulations, and 90 % confidence intervals.

Initial adoption rate [%]	Usage stop probability [%]	PDR	Maximum share of Infectious individuals [%]	Time till maximum share of infectious individuals	Duration of the pandemic [days]	Average time of quarantine per person [days]	Share of susceptible quarantine time [%]	Share of susceptible people at the end [%]	Overall R_0 value []
0	0	No CT	55.796 ± 0.145	27.367 ± 64.173	72.500 ± 1.415	14.690 ± 0.025	0.000 ± 0.000	7.511 ± 0.103	2.799 ± 0.012
20	0	0.2 m	55.659 ± 0.232	26.633 ± 55.641	74.800 ± 1.798	14.722 ± 0.028	0.084 ± 0.006	7.585 ± 0.095	2.790 ± 0.011
20	0	1 m	54.545 ± 0.229	26.900 ± 57.692	76.200 ± 1.524	15.019 ± 0.028	1.046 ± 0.032	8.165 ± 0.084	2.728 ± 0.009
20	0	2 m	52.069 ± 0.267	27.167 ± 66.745	76.533 ± 1.547	15.432 ± 0.031	3.209 ± 0.067	9.130 ± 0.109	2.634 ± 0.010
20	0	10 m	50.169 ± 0.210	27.533 ± 73.458	77.467 ± 1.604	15.818 ± 0.032	5.722 ± 0.099	9.979 ± 0.118	2.560 ± 0.010
20	0	Sites-wide	46.565 ± 0.278	29.667 ± 90.212	81.667 ± 2.232	21.899 ± 0.199	23.636 ± 0.499	14.500 ± 0.155	2.258 ± 0.008
40	0	0.2 m	55.301 ± 0.235	26.733 ± 60.532	73.433 ± 1.556	14.829 ± 0.028	0.366 ± 0.018	7.793 ± 0.090	2.767 ± 0.010
40	0	1 m	50.846 ± 0.277	27.500 ± 62.879	80.567 ± 1.501	15.837 ± 0.030	4.478 ± 0.086	9.823 ± 0.122	2.573 ± 0.011
40	0	2 m	43.738 ± 0.303	28.333 ± 83.640	84.500 ± 2.153	17.111 ± 0.035	11.499 ± 0.151	12.227 ± 0.132	2.394 ± 0.009
40	0	10 m	40.453 ± 0.310	30.233 ± 94.238	86.167 ± 2.085	18.173 ± 0.045	17.736 ± 0.227	14.291 ± 0.154	2.269 ± 0.008
40	0	Sites-wide	34.066 ± 0.333	33.767 ± 128.989	87.767 ± 2.356	31.920 ± 0.584	44.787 ± 0.734	26.254 ± 0.255	1.813 ± 0.007
60	0	0.2 m	54.714 ± 0.285	26.867 ± 57.892	74.133 ± 1.208	15.030 ± 0.031	0.979 ± 0.037	8.187 ± 0.122	2.726 ± 0.013
60	0	1 m	44.276 ± 0.317	27.767 ± 53.548	92.100 ± 3.621	17.206 ± 0.039	10.676 ± 0.123	12.306 ± 0.151	2.389 ± 0.010
60	0	2 m	32.488 ± 0.277	32.267 ± 145.062	100.533 ± 3.211	19.700 ± 0.070	24.091 ± 0.210	17.828 ± 0.194	2.098 ± 0.008

Initial adoption rate [%]	Usage stop probability [%]	PDR	Maximum share of Infectious individuals [%]	Time till maximum share of infectious individuals	Duration of the pandemic [days]	Average time of quarantine per person [days]	Share of susceptible quarantine time [%]	Share of susceptible people at the end [%]	Overall R_0 value []
60	0	10 m	30.678 ± 0.386	35.800 ± 116.680	102.300 ± 2.536	22.138 ± 0.154	35.126 ± 0.497	22.292 ± 0.276	1.931 ± 0.009
60	0	Sites-wide	19.478 ± 0.283	42.567 ± 216.113	107.033 ± 3.411	51.062 ± 1.541	65.887 ± 0.754	45.340 ± 0.299	1.445 ± 0.004
80	0	0.2 m	53.281 ± 0.280	26.900 ± 53.360	77.600 ± 1.269	15.380 ± 0.036	2.146 ± 0.055	8.843 ± 0.105	2.661 ± 0.010
80	0	1 m	34.515 ± 0.328	27.933 ± 67.127	106.900 ± 2.958	19.229 ± 0.066	20.137 ± 0.191	16.458 ± 0.261	2.160 ± 0.012
80	0	2 m	19.865 ± 1.624	38.667 ± 235.762	132.967 ± 9.737	23.486 ± 1.825	47.843 ± 3.045	33.764 ± 5.273	1.683 ± 0.066
80	0	10 m	14.144 ± 1.179	41.867 ± 323.817	139.800 ± 11.154	30.284 ± 2.369	65.305 ± 2.397	43.879 ± 4.518	1.483 ± 0.053
80	0	Sites-wide	2.810 ± 0.421	62.167 ± 769.469	150.200 ± 16.753	94.284 ± 12.250	90.813 ± 1.178	82.570 ± 2.286	1.070 ± 0.033
100	0	0.2 m	52.227 ± 0.292	26.933 ± 68.895	81.033 ± 1.803	15.793 ± 0.039	3.627 ± 0.076	9.744 ± 0.112	2.580 ± 0.010
100	0	1 m	21.385 ± 0.396	27.667 ± 93.485	164.467 ± 6.733	23.831 ± 0.162	36.597 ± 0.341	21.732 ± 0.403	1.951 ± 0.014
100	0	2 m	2.391 ± 0.386	37.900 ± 738.780	95.767 ± 13.384	13.404 ± 2.720	85.156 ± 0.757	89.891 ± 2.340	1.026 ± 0.025
100	0	10 m	1.059 ± 0.190	20.933 ± 516.769	56.200 ± 6.819	7.553 ± 1.616	93.846 ± 0.369	97.683 ± 0.574	0.928 ± 0.022
100	0	Sites-wide	0.791 ± 0.149	13.900 ± 43.419	41.733 ± 1.896	18.326 ± 1.421	98.489 ± 0.242	98.823 ± 0.224	0.885 ± 0.020
60	25	0.2 m	54.714 ± 0.285	26.867 ± 57.892	74.633 ± 1.264	15.031 ± 0.031	0.979 ± 0.038	8.156 ± 0.128	2.729 ± 0.013
60	25	1 m	44.342 ± 0.302	27.800 ± 55.052	91.167 ± 2.817	17.141 ± 0.040	10.308 ± 0.117	11.901 ± 0.148	2.416 ± 0.010
60	25	2 m	32.784 ± 0.319	32.733 ± 145.475	100.033 ± 2.699	19.507 ± 0.053	21.633 ± 0.177	14.079 ± 0.165	2.281 ± 0.009

Initial adoption rate [%]	Usage stop probability [%]	PDR	Maximum share of Infectious individuals [%]	Time till maximum share of infectious individuals	Duration of the pandemic [days]	Average time of quarantine per person [days]	Share of susceptible quarantine time [%]	Share of susceptible people at the end [%]	Overall R_0 value []
60	25	10 m	33.499 ± 0.390	36.767 ± 108.477	103.767 ± 2.251	21.227 ± 0.074	28.903 ± 0.277	15.151 ± 0.190	2.224 ± 0.010
60	25	Sites-wide	35.853 ± 0.853	41.500 ± 177.638	111.567 ± 3.488	33.797 ± 0.260	42.756 ± 0.651	16.801 ± 0.341	2.145 ± 0.016
60	50	0.2 m	54.714 ± 0.285	26.867 ± 57.892	75.233 ± 1.313	15.034 ± 0.032	0.977 ± 0.038	8.145 ± 0.134	2.730 ± 0.014
60	50	1 m	44.245 ± 0.309	27.700 ± 51.526	89.867 ± 2.140	17.088 ± 0.045	10.126 ± 0.111	11.687 ± 0.161	2.431 ± 0.011
60	50	2 m	33.039 ± 0.342	32.867 ± 141.664	95.767 ± 2.093	19.269 ± 0.047	20.228 ± 0.138	11.959 ± 0.149	2.412 ± 0.010
60	50	10 m	35.766 ± 0.523	37.100 ± 97.273	98.900 ± 2.402	20.572 ± 0.048	25.934 ± 0.229	11.857 ± 0.120	2.418 ± 0.008
60	50	Sites-wide	44.312 ± 0.790	39.400 ± 116.474	96.800 ± 2.635	26.408 ± 0.086	37.088 ± 0.431	11.010 ± 0.192	2.480 ± 0.015
60	75	0.2 m	54.717 ± 0.286	26.867 ± 57.892	75.100 ± 1.496	15.029 ± 0.033	0.976 ± 0.038	8.169 ± 0.129	2.728 ± 0.013
60	75	1 m	44.278 ± 0.312	27.800 ± 52.822	90.167 ± 2.324	17.052 ± 0.052	9.931 ± 0.099	11.485 ± 0.204	2.445 ± 0.014
60	75	2 m	33.203 ± 0.367	33.300 ± 144.067	96.467 ± 1.749	19.122 ± 0.039	19.418 ± 0.147	10.375 ± 0.118	2.528 ± 0.009
60	75	10 m	37.526 ± 0.614	37.033 ± 93.211	91.567 ± 1.598	20.130 ± 0.037	24.541 ± 0.185	10.147 ± 0.139	2.546 ± 0.011
60	75	Sites-wide	48.984 ± 0.609	38.033 ± 111.968	87.600 ± 1.878	23.205 ± 0.057	33.234 ± 0.289	8.807 ± 0.098	2.664 ± 0.009
60	100	0.2 m	54.718 ± 0.286	26.867 ± 57.892	74.600 ± 1.427	15.026 ± 0.032	0.975 ± 0.038	8.192 ± 0.126	2.725 ± 0.013
60	100	1 m	44.259 ± 0.324	27.700 ± 54.365	92.200 ± 2.039	17.044 ± 0.037	9.782 ± 0.107	11.175 ± 0.164	2.467 ± 0.012
60	100	2 m	33.543 ± 0.439	33.033 ± 148.047	93.100 ± 2.244	18.970 ± 0.037	19.014 ± 0.119	9.903 ± 0.129	2.566 ± 0.011

Initial adoption rate [%]	Usage stop probability [%]	PDR	Maximum share of Infectious individuals [%]	Time till maximum share of infectious individuals	Duration of the pandemic [days]	Average time of quarantine per person [days]	Share of susceptible quarantine time [%]	Share of susceptible people at the end [%]	Overall R_0 value []
60	100	10 m	38.548 ± 0.731	37.567 ± 90.660	90.233 ± 1.596	19.875 ± 0.033	23.780 ± 0.177	9.044 ± 0.106	2.642 ± 0.010
60	100	Sites-wide	52.271 ± 0.422	37.233 ± 113.356	82.667 ± 1.407	21.641 ± 0.041	30.365 ± 0.190	7.834 ± 0.077	2.763 ± 0.009

References

1. German Federal Office of Statistics. Bevölkerung und Erwerbstätigkeit, Haushalte und Familien, Ergebnisse des Mikrozensus 2019 [Available from: https://www.destatis.de/DE/Themen/Gesellschaft-Umwelt/Bevoelkerung/Haushalte-Familien/Publikationen/Downloads-Haushalte/haushalte-familien-2010300197004.pdf?__blob=publicationFile.
2. Liu Q-H, Ajelli M, Aleta A, Merler S, Moreno Y, Vespignani A. Measurability of the epidemic reproduction number in data-driven contact networks. Proceedings of the National Academy of Sciences. 2018;115(50):12680-5.
3. Federal Statistical Office of Germany. Erwerbstätige Inländer - Statistisches Bundesamt 2020 [Available from: https://www.destatis.de/DE/Themen/Wirtschaft/Konjunkturindikatoren/Arbeitsmarkt/karb811.html.
4. Federal Statistical Office of Germany. Bildung und Kultur, Allgemeinbildende Schulen, Schuljahr 2019/2020 2020 [Available from: https://www.destatis.de/DE/Themen/Gesellschaft-Umwelt/Bildung-Forschung-Kultur/Schulen/Publikationen/Downloads-Schulen/allgemeinbildende-schulen-2110100207005.xlsx?__blob=publicationFile.
5. Federal Statistical Office of Germany. Bildung und Kultur, Studierende an Hochschulen, Wintersemester 2019/2020 2020 [Available from: https://www.destatis.de/DE/Themen/Gesellschaft-Umwelt/Bildung-Forschung-Kultur/Hochschulen/Publikationen/Downloads-Hochschulen/studierende-hochschulen-endg-2110410207004.pdf?__blob=publicationFile.
6. Federal Ministry for Family Affairs, Senior Citizens,, Women and Youth,. 790.000 Betreuungsplätze für Kinder unter drei Jahren 2019 [Available from: https://www.bmfsfj.de/bmfsfj/aktuelles/alle-meldungen/790-000-betreuungsplaetze-fuer-kinder-unter-drei-jahren/138140.
7. Nielsen. Nielsen consumers deutschland 2018 [Available from: https://www.nielsen.com/wp-content/uploads/sites/3/2019/08/Nielsen-Consumers-2018-Deutschland_PDF.pdf.

8. Li Q, Guan X, Wu P, Wang X, Zhou L, Tong Y, et al. Early transmission dynamics in Wuhan, China, of novel coronavirus–infected pneumonia. New England Journal of Medicine. 2020.
9. He X, Lau EH, Wu P, Deng X, Wang J, Hao X, et al. Temporal dynamics in viral shedding and transmissibility of COVID-19. Nature medicine. 2020;26(5):672-5.
10. Beldomenico PM. Do superspreaders generate new superspreaders? a hypothesis to explain the propagation pattern of COVID-19. International Journal of Infectious Diseases. 2020.
11. Liu Y, Gayle AA, Wilder-Smith A, Rocklöv J. The reproductive number of COVID-19 is higher compared to SARS coronavirus. Journal of travel medicine. 2020.
12. Hethcote HW, Tudor DW. Integral equation models for endemic infectious diseases. Journal of mathematical biology. 1980;9(1):37-47.
13. Li MY, Muldowney JS. Global stability for the SEIR model in epidemiology. Mathematical biosciences. 1995;125(2):155-64.
14. Contact tracing simulation of different proximity detection ranges and usage stops for COVID-19 2020 [Available from: https://github.com/kpandl/COVID-19-contact-tracing-simulation.
